# Relationship troubles at the mitochondrial level and what it might mean for human disease

**DOI:** 10.1098/rsob.240331

**Published:** 2025-05-21

**Authors:** Rachel James

**Affiliations:** ^1^ University of Edinburgh, Edinburgh, UK

**Keywords:** mitochondria, genome, eukaryotic cell, endosymbiosis, human disease

## Abstract

Understanding and treating disease depend upon our knowledge of how the body works. The biomedical approach to disease describes health purely in terms of biological factors, with a focus on the genome as the molecular basis for cellular function and dysfunction in disease. However, the eukaryotic cell has evolved as a partnership between prokaryotic cells with mitochondria being crucial to this relationship. Aside from their role as bioenergetic and biosynthetic hubs, mitochondria are also involved in cell signalling and cell fate pathways, playing a multifaceted role in cell function and health. Crucially, mitochondria are implicated in most diseases. Perhaps then, visualizing biomedical function on the backdrop of endosymbiosis may provide another viewpoint for explaining and treating disease.

**Figure:**
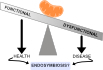

## 1. Introduction

Understanding and treating disease depend upon our knowledge of how the body works. The biomedical approach to disease dominates the Western approach to medicine, breaking the body down into identify the biological parts that need ‘fixing’. Health is mostly described in biological terms, with a persistent focus on the nuclear genome as the molecular basis for cellular function and subsequent dysfunction in disease. This is logical, in that the genome contains the genetic information needed to encode the proteins that enable our cells to function throughout our lifespan. Yet the cells in our body have evolved as a partnership between single-celled organisms, with the nuclear genome being only one of several outcomes of this relationship. These evolutionary innovations did not appear in our cells fully formed and ready for use, they have evolved and been shaped to perform specific cellular functions. On either side of this partnership are the nucleus and mitochondria.

## 2. The pivotal role of mitochondria in disease

Altered mitochondrial function is implicated in many, if not all, human diseases, encompassing all cell types and organs of the body and all stages of life (examples reviewed in [1–5]). Of the World Health Organization’s ‘top-10’ causes of global disease [6], mitochondrial dysfunction can be implicated in all 10. Several of these diseases have long been associated with mitochondrial dysfunction (e.g. ischemic heart disease [7], stroke [8] and Alzheimer’s disease [9]), while others are less commonly linked to mitochondria (e.g. COVID-19 [10], chronic obstructive pulmonary disease [11], diabetes mellitus [12] and tuberculosis [13]). That these diseases are etiologically distinct yet in some capacity have mitochondrial dysfunction as a pathological hallmark, raises the possibility that understanding shared mitochondrial mechanisms of disease could lead to mitochondrial-based treatments that are efficacious across distinct diseases.

Popularized as the ‘powerhouse of the cell’ [14], mitochondria are well known as the bioenergetic core of the cell. As arbitrators of health and disease, they are ideally situated within the cell, operating at an interface between the internal and external environments. Mitochondria are acutely sensitive to stressors that originate both inside (e.g. damaged or misfolded proteins) and outside (e.g. antibiotics, pesticides and diet) the cell [15]. Somewhat counter-intuitively, a small amount of mitochondrial stress is beneficial for cellular resilience, while too much results in malfunctioning mitochondria that can tip the cell into a pathological state [16–18].

For many diseases, early, prenatal development can influence health and disease vulnerability in later life [19]. It is well known that the mitochondrial genome is inherited through the maternal oocyte at the start of development. Somewhat overlooked is that mitochondria themselves are inherited. As mitochondria cannot be made de novo, new mitochondria must divide from pre-existing ones (through a bacterial-like process of division known as binary fission) [20]. This means that the mitochondrial population for the entire human body is derived from a single starting population inherited through the oocyte [21,22]. The health of these maternally inherited mitochondria is therefore critical for organismal health. With their increasingly recognized role in cell signalling [23] and cell fate [24], mitochondria could feasibly represent a common cellular pathway for the transmission of early developmental effects across a spectrum of disorders. It is no wonder then, that our health suffers if mitochondrial function is compromised.

Nevertheless, the prevalent opinion of mitochondria posits them as compliant organelles under nuclear control, muddying somewhat that they did not appear within the cell as passive bystanders, obediently shuttling around the cell to generate energy for their host. Mitochondria exist within the cell through a long-standing relationship that is so embedded in the fabric of the eukaryotic cell that it often remains hidden. To recognize this contribution, it is necessary to go back to their origin as one side of the prokaryotic partnership that lies at the heart of eukaryotic cells.

## 3. Endosymbiosis and the generation of eukaryotic cells

All living organisms can be sorted into one of the two groups depending on the fundamental structure of their cells [25]. Prokaryotes are typically single-celled organisms, made of cells that lack a nucleus or membrane-bound compartments (or organelles). They can be subdivided into two groups: bacteria and archaea. In contrast, eukaryotes encompass all multicellular organisms including animals, plants and fungi. The eukaryotic cell is believed to have emerged around 1.5−1.8 billion years ago [26,27]. The exact details of how this happened are uncertain. Nevertheless, there is little doubt that mergers between prokaryotic cells played a central role. Of these, the acquisition of an ancient bacterium by a host prokaryote was a defining moment for eukaryotes. The evolutionary biologist Lynn Margulis brought this hypothesis on the endosymbiotic theory of eukaryotic cells to mainstream attention in 1967 [28]. The main tenet of this theory states that mitochondria were once free-living bacteria (an alpha-proteobacterium) that merged in some manner with another host prokaryotic cell (possibly an archaeal species), leading to the generation of a new cell type: the eukaryotic cell. Symbiosis is defined as any close long-term biological interaction between two different biological organisms [29]; endosymbiosis is the closet of all relationships, with one organism literally living inside another. Prokaryotic mergers are rare in nature [30], making the endosymbiotic relationship at play inside our cells ever more intriguing.

## 4. Mitochondria and the emerging eukaryotic cell

**Figure 1.**
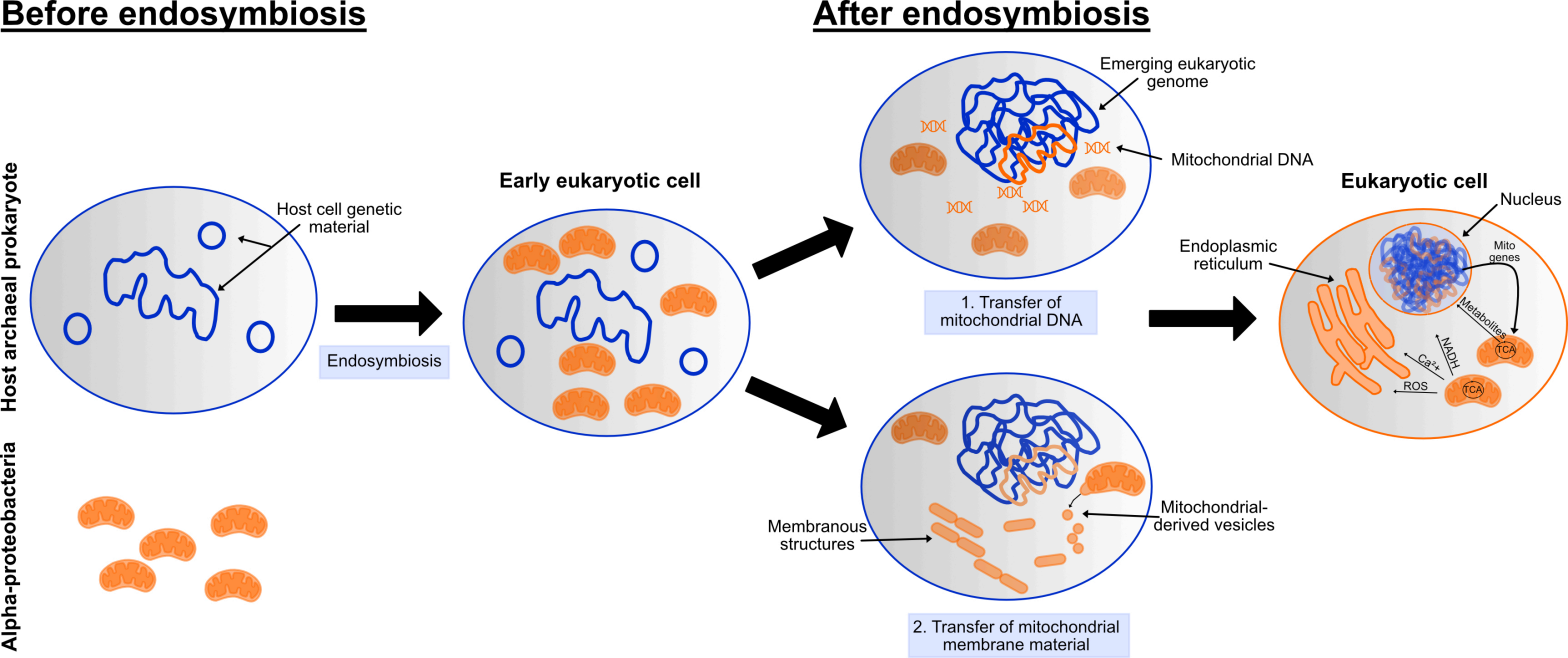
Mitochondria and the emerging eukaryotic cell. The endosymbiotic theory of eukaryotic cells states that free-living bacteria (alpha-proteobacteria) merged with another prokaryotic cell (possibly an archaeal species) to generate the eukaryotic cell. In the early eukaryotic cell, it is likely that the genetic material of the host prokaryote was arranged in cytoplasmic forms. Following endosymbiosis, the mitochondrial genome was transferred to the host prokaryotic genome. With increasing evolutionary time, the centrally housed eukaryotic genome emerged (‘1. Transfer of mitochondrial DNA’). In addition, mitochondrial membrane material (particularly lipids) was incorporated into eukaryotic cell membranes. The generation and fusion of mitochondrial-derived vesicles likely played a role in development of intracellular membranous structures (‘2. Transfer of mitochondrial membrane material’). In modern-day eukaryotic cells, these membrane structures have evolved into the endomembrane system, which includes the nuclear envelope and endoplasmic reticulum. Mitochondria communicate extensively within the eukaryotic cell using a variety of signalling molecules (e.g. NADH/NAD^+^, calcium (Ca^2+^) and reactive oxygen species (ROS)), some of which are generated as by-products of the tricarboxylic acid cycle (TCA). The nuclear genome now contains most of the genes needed for mitochondrial function (Mito genes). The eukaryotic cell shown is a simplified version for the purposes of this perspective. In all cartoons, host cellular components are shown in blue, with mitochondrial components shown in orange. However, their depiction in a single colour is for ease of illustration only, and these cellular components are more often a mix of host and mitochondrial contribution. Also, note that the schematic provides a very broad overview of one likely scenario. There are alternative viewpoints and hypotheses for the emergence of eukaryotic cells.

Although our definition of prokaryotes and eukaryotes has been based on the absence (pro) or presence (eu) of a nucleus (karyote), this masks somewhat that the nucleus, and its incumbent eukaryotic genome, are only one of several eukaryotic innovations [27]. Aside from a nucleus with linear chromosomes, eukaryotic cells can be distinguished from prokaryotes by a mostly larger cell size, the presence of membrane-bound organelles (including mitochondria) and distinct molecular mechanisms (such as transcription/translation, autophagy). These innovations have likely materialized in evolutionary fits and bursts on the backdrop of this endosymbiotic relationship (figure 1). That these features have evolved is key here. Based upon the cell structure of prokaryotes, the eukaryotic cell of modern eukaryotes (i.e. all human cells) is unlikely to be what it was like at the time of the initial prokaryotic merger. For example, the genetic material of early eukaryotes was likely organized in cytoplasmic forms (in circular chromosomes and plasmids), akin to prokaryotes. Housing the genetic material in a central nuclear compartment evolved in eukaryotic cells [31,32]. Likewise, the refinement of distinct subcellar compartments for specific cellular functions was adapted and modified in the emerging eukaryotic cell [33,34]. These changes presumably occurred alongside the bacteria-to-mitochondria transition. Therefore, understanding these subcellular transitions may provide insight into how mitochondrial function can influence disease.

### 4.1. Mitochondrial contribution to the eukaryotic genome

Lynn Margulis and others were able to connect the dots between mitochondria and bacteria based upon morphological similarities. Put simply, the shape and dynamism of mitochondria within the cell are strikingly similar to those of their bacterial counterparts. Nonetheless, there are differences between mitochondria and bacteria that hint at the evolutionary adaptations required to convert free-living alpha-proteobacteria into host-resident mitochondria. One well-documented adaptation is transfer of the mitochondrial genome to the emerging nuclear genome [35]. Gene transfer to the host genome is a well-described characteristic in co-evolution of other host-restricted endosymbionts [36,37]. Because of this transfer, the eukaryotic genome is basically a chimera of prokaryotic genetic material [38], comprised of the host archaeon genome, mitochondrial genome and genetic material from other prokaryotes (additional bacterial and archaeal species; viruses). This original DNA has since been duplicated, deleted, shuffled and recombined in zillions of different ways, contributing to the increased size and complexity of the eukaryotic genome [39]. Modern-day eukaryotes, including humans, show remnants of this mitochondrial gene exodus in the nuclear genome. With a central role of mitochondria in cell metabolism, it is perhaps unsurprising that metabolic genes are generally thought to be of mitochondrial origin, while genes involved in transcription and translation are often archaeal derivatives [40]. From a biomedical perspective then, it is interesting to speculate that a gene that now localizes to the nucleus or cytoplasm could be mitochondrial in origin. Further mitochondrial imprints in the nuclear genome are evidenced by the presence of non-expressed fragments of nuclear mitochondrial DNA segments (NUMTs) and a specific type of intron. NUMTs are scattered throughout the genome and have been linked to diseases such as cancer [41,42]. Some NUMTs are common among humans, while rarer ones suggest that the transfer of mitochondrial DNA to the nuclear genome is still ongoing [43]. The arrival of introns in the eukaryotic genome set the foundations for RNA splicing and the ensuing increase in protein diversity, a prerequisite for the expanding phenotypic variation and multicellularity of eukaryotic organisms. Although not the only source of intronic sequence in the nuclear genome, one class of introns are thought to have originated from early transfer of the mitochondrial genome to the nuclear genome [44]. Again, these have been linked to several diseases in humans [45].

### 4.2. Mitochondrial contribution to the endomembrane system

The eukaryotic endomembrane system includes the endoplasmic reticulum, Golgi body, vesicles (such as lysosomes and peroxisomes), nuclear envelope and plasma membrane. Each membranous compartment has evolved to perform a specific function, predominantly of protein or lipid biology. Although the exact means by which the endomembrane system arose is under debate [34,46,47], there is agreement that along with an abundant supply of genetic material, the original mitochondrial endosymbiont also provided the early eukaryotic cell with membrane material. This implicates mitochondrial endosymbiosis in evolution of not only the nucleus and eukaryotic genome but also the endomembrane system. Indeed, the incorporation of bacterial-like phospholipids in eukaryotic membranes [48] has led to a so-called ‘lipid divide’ that can distinguish bacterial and eukaryotic membranes from archaea [49]. Related to this is the universal use of vesicles for prokaryotic communication [50]. For example, Gram-negative bacteria (which include the mitochondrial lineage) use vesicles to interact with other bacterial species and the environment [51]. Inside the emerging eukaryotic cell, the release of vesicles from early mitochondria is thought to have fused into membranous structures that have now evolved into the highly specialized and delineated endomembrane system [34,47]. Feasibly then, the endomembrane system may reflect another adaptation of the host cell to mitochondrial inhabitation, either to bolster the bioenergetic capabilities of mitochondria (e.g. by sequestering biochemical functions in the endoplasmic reticulum) or to contain their damage (e.g. using lysosomes to remove damaged or dysfunctional mitochondria). Interestingly, mitochondria have recently been shown to shed mitochondrial-derived vesicles [52–54], suggesting preservation of this ancient communication method within the modern-day eukaryotic cell. Like the involvement of mitochondrial genomic remnants in disease, these vesicles are increasingly associated with pathology in several diseases [55].

## 5. Mitochondria are more than an energy supply for the eukaryotic cell

Because of mitochondrial gene transfer to the nuclear genome, the human mitochondrial genome now contains only 37 genes [56]. This is enough to encode the bare minimum required for oxidative phosphorylation and ensure mitochondrial primacy within this domain. The remaining approximately 1000−1500 proteins [57] needed for mitochondrial function are encoded by the nuclear genome, translated into proteins and then transported into mitochondria. One of the main functional consequences following the emergence of the nucleus in eukaryotic cells was partitioning of the genomic DNA into a separate compartment, allowing uncoupling of transcription (nucleus) and translation (cytoplasm). With outsourcing of the mitochondrial genome to the nucleus, mitochondria are now functionally reliant on both of these processes for efficient function. For endosymbiosis to have taken hold, in a presumably reciprocal manner, communication and cooperation between the original prokaryotes must have arisen at some point, either as an inevitable consequence or enforced by one or other of the partners. It is easy to envision the nucleus and eukaryotic genome on one side of the endosymbiotic partnership: providing a readily available source of DNA for evolution and the building blocks of proteins for cellular function. On the other side, mitochondria may be just as instrumental a partner.

Mitochondria are well known for their role in oxidative phosphorylation and biosynthesis [58]. Their ability to supply early eukaryotes with a readily available source of ATP fostered the development and refinement of energy-intensive functions for eukaryotic cell function. However, mitochondria are increasingly recognized for their involvement in other cellular functions. These are often intimately linked to their energetic and/or metabolic capabilities but not intuitively associated with mitochondria. One of the first indications that mitochondria were doing something else within the cell came from work showing that a peptide derived from an oxidative phosphorylation protein encoded in mitochondrial DNA could prime the immune system against bacterial infections [59,60]. This early work highlighted the role of mitochondria in the innate immune system. The subsequent identification of mitochondria as the subcellular trigger for apoptosis [61] further bolstered their involvement in non-bioenergetic cellular functions. Since these early findings, mitochondrial involvement in other cell fate processes (such as cell differentiation [62–65], cell cycle control [66–68] and pluripotency [69,70]) has been experimentally demonstrated across a variety of bodily systems and cell types. Despite original assumptions, their role in cell fate does not always depend on their energy-producing capabilities [71], and their ability to supply the cell with ATP may not be the only reason for their involvement in these processes. At the same time, their role as signalling organelles in extensive networks of cellular function has gained widespread traction. As metabolic hubs, mitochondria are the source of various signalling molecules [72,73] and can act as signalling platforms [74], enabling interaction with the nucleus, other organelles and more externally facing pathways (e.g. immune signalling). Their signalling capability allows mitochondria to communicate within and between cells, providing a mechanistic basis for their role in cell fate. Bacteria are highly communicative, using various molecular and chemical mediators [75,76] and physical intermediates (such as electricity [77] and mechanical force [78]) to transmit information within and across species. Therefore this role in cell signalling is consistent with their microbial ancestry, and there is no reason to presume that this communication was squashed entirely in the eukaryotic cell. Indeed, the importance of mitochondrial-derived vesicles in prokaryotic communication [51] and the emerging eukaryotic cell [34,47] has already been introduced. Early communication between the incoming alpha-proteobacteria and host prokaryote may imaginably be similar to strategies used by bacterial pathogens to gain host control during infection [79,80] or in other cases of symbiosis where communication is rapidly established between the host and endosymbiont [29].

The formation of mitochondrial communication networks between the emerging genome and other cellular functions must have been essential for the bacteria-to-mitochondria transition. Of all these networks, perhaps the mitochondrion’s role in epigenomic regulation may be most pertinent here [81]. The tricarboxylic acid cycle (TCA) at the heart of mitochondria generates the metabolic intermediates for modifying several epigenetic marks, especially histone acetylation and methylation, and DNA methylation [82,83]. The TCA cycle is evolutionarily ancient [84]; therefore, presumably mitochondria and the original alpha-proteobacteria have always had the means to manipulate the emerging eukaryotic genome. For endosymbiosis and the evolution of eukaryotic cells, mitochondrial energy was essential but they have contributed more than an energy supply. Their capacity to communicate with the cell and influence cell fate may arguably have been just as pivotal in shaping eukaryotic cell function.

## 6. Endosymbiosis and human disease

**Figure 2.**
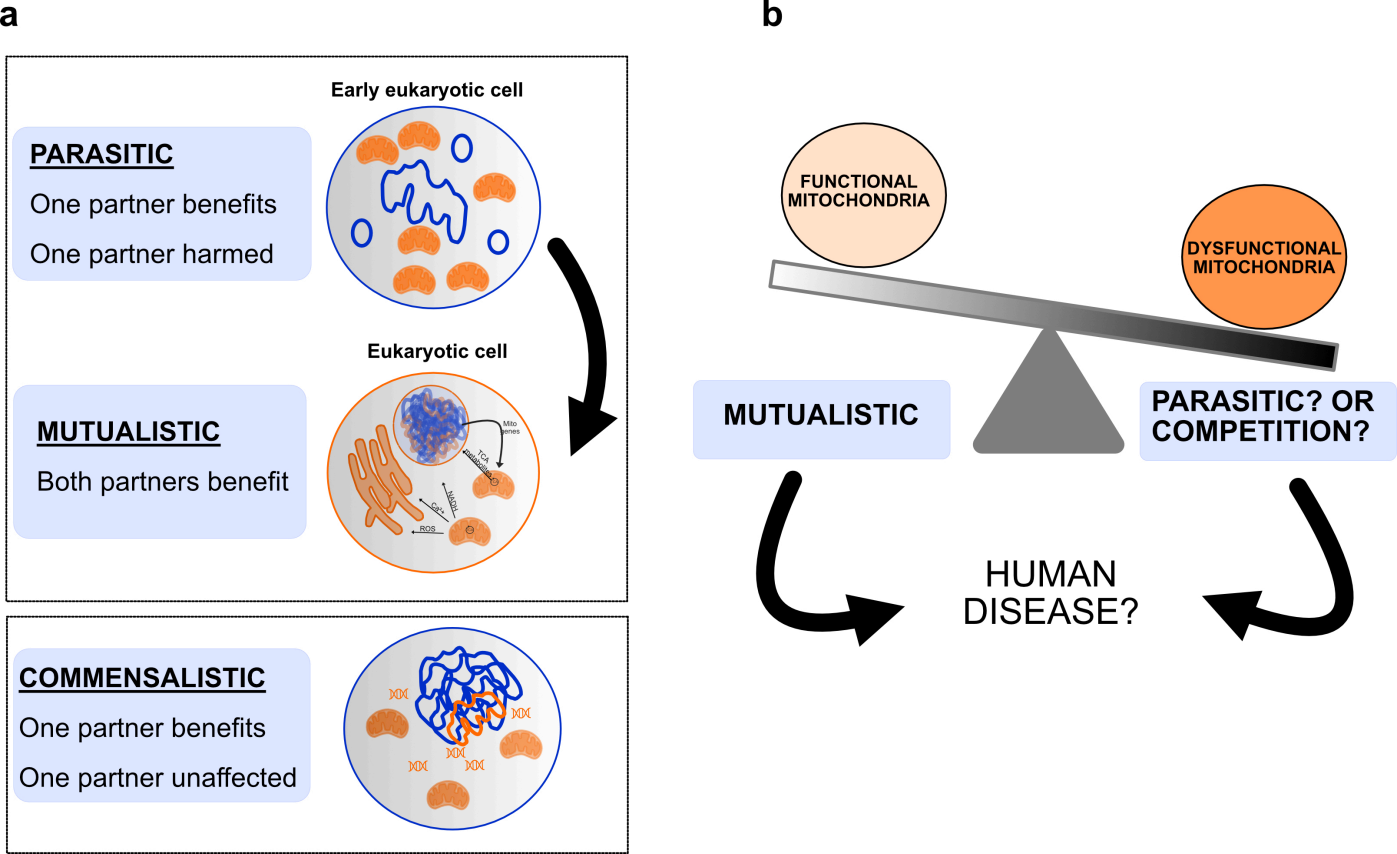
Harnessing endosymbiosis. (a) There are different types of symbiotic relationships. The endosymbiotic relationship of eukaryotic cells is thought to be mutualistic, with both original prokaryotes benefiting—early mitochondria provided a ready source of energy and nutrients for the host cell, and the original host prokaryote provided protection, a mode of transport and food for mitochondria. It is feasible, however, that in the early eukaryotic cell, this relationship may have been parasitic. In this scenario, early mitochondrial endosymbionts may have been harmful to the host cell, perhaps causing cell death. Stabilization of this relationship is likely to have occurred following mutual adaptation of mitochondrial endosymbionts to the host cell environment. Although it is harder to envision a communalistic relationship for this partnership, whereby one partner is truly unaffected by the other, potentially this occurred at stages during evolution of modern-day eukaryotes. (b) In human cells, mitochondria exist in a variable state of function/dysfunction. Various internal and external triggers can impact the health of mitochondria. It is unclear what impact this will have on the mutualistic endosymbiotic relationship. Potentially, it may shift the relationship towards a different form (e.g. parasitic) or cause competition between the original prokaryotic partners. Understanding the underpinnings of this relationship may help identify therapies for disease.

But why on earth does this billion-year-old relationship matter to human disease? Despite the importance of mitochondria to cell function, little is known about how they are stably maintained within the cell. Yet no successful relationship is ever one-sided, and this is as true for endosymbiosis as any other. Symbiotic relationships take different forms depending somewhat on the power dynamic within the partnership. In eukaryotic cells this relationship is thought to be mutualistic [85], with both original prokaryotes benefiting–the early mitochondrion provided a ready source of energy and nutrients for the host cell, while in turn, the original host provided protection, a mode of transport and food for the mitochondrion (figure 2). Both have been invested in each other’s long-term survival. But relationships change [86,87], and it is not inconceivable that the endosymbiotic relationship has changed over time, and indeed, could still change or be changing.

The closest free-living ancestor of mitochondria is currently purported to be *Rickettsia prowazekii* [88], a pathogenic intracellular bacterium that causes epidemic typhus. Although mitochondria have been shown to move between cells in the laboratory [89,90], they are not pathogenic and do not appear to harm their hosts in a normal state of function. Nevertheless, considering their putative ancestry, it is not hard to envision that at some stage, the relationship between the host prokaryote and early mitochondrion could have been parasitic. In speculative support of this, mitochondria do not have a cell wall, a characteristic of their ancestral lineage as alpha-proteobacteria. Cell wall shedding is a strategy adopted by certain species of pathogenic bacteria to evade intracellular detection by the host [91]. Hypothetically, the lack of a mitochondrial cell wall may reflect an adaptation of the original mitochondrion to the host cell and could be one way in which a free-living bacterium has survived long-term inside another organism. Further, while the acquisition of an alpha-proteobacterium (as the mitochondrial ancestor) was a pivotal step towards the generation of eukaryotes, there are eukaryotic organisms that no longer contain mitochondria [92]. These were originally thought to have evolved without mitochondria but have since been shown to have lost them at some point during their evolution. So, mitochondria are not necessarily a fixed, immutable feature of our cells. It is not inconceivable that the endosymbiotic relationship may wax and wane with time, perhaps reflecting changes both inside and outside the cell.

## 7. Conclusions

Considering all that is known about mitochondria as heritable, biosynthetically versatile stress sensors with a fundamental role in the very fabric of our cells, it is not unreasonable to say they have contributed more than their fair share to eukaryotic cell function: their genome and membranes, the building blocks for gene regulation and cellular signalling networks. All the while churning out energy and metabolites to keep the cell alive. With their apparently universal involvement in human disease, perhaps another way of explaining, and in turn even treating disease, could be to imagine cellular pathology as a breakdown in this endosymbiotic relationship. Rather than neglecting this highly successful relationship, understanding how to maintain its collaborative nature and harness all facets of the mitochondrial power within our cells may be crucial for long-lasting human health.

## Data Availability

This article has no additional data.
